# Narrowband Spontaneous Emission Amplification from a Conjugated Oligomer Thin Film

**DOI:** 10.3390/polym12010232

**Published:** 2020-01-17

**Authors:** Mohamad S. AlSalhi, Mamduh J. Aljaafreh, Saradh Prasad

**Affiliations:** 1 Department of Physics and Astronomy, College of Science, King Saud University, 11451 Riyadh, Saudi Arabia; malsalhi@ksu.edu.sa (M.S.A.); maljaafreh@ksu.edu.sa (M.J.A.); 2 Research Chair on laser diagnosis of cancers, Department of Physics and Astronomy, College of Science, King Saud University, 11451 Riyadh, Saudi Arabia

**Keywords:** thin film, dimer, amplified spontaneous emission (ASE), time-resolved spectroscopy (TRS)-conjugated oligomer (BECV-DHF)

## Abstract

In this paper, we studied the laser and optical properties of conjugated oligomer (CO) 1,4-bis(9-ethyl-3-carbazo-vinylene)-9,9-dihexyl-fluorene (BECV-DHF) thin films, which were cast onto a quartz substrate using a spin coating technique. BECV-DHF was dissolved in chloroform at different concentrations to produce thin films with various thicknesses. The obtained results from the absorption spectrum revealed one sharp peak at 403 nm and two broads at 375 and 428 nm. The photoluminescence (PL) spectra were recorded for different thin films made from different concentrations of the oligomer solution. The threshold, laser-induced fluorescence (LIF), and amplified spontaneous emission (ASE) properties of the CO BECV-DHF thin films were studied in detail. The ASE spectrum was achieved at approximately 482.5 nm at a suitable concentration and sufficient pump energy. The time-resolved spectroscopy of the BECV-DHF films was demonstrated at different pump energies.

## 1. Introduction

Among the several types of organic semiconductor materials, conjugated materials have been subjected to intensive research studies for many years [1,2,3,4,5,6,7,8,9]. There has been widespread interest in conjugated materials for applications in optoelectronic devices, such as chemical sensors [10], light-emitting diodes [4,7,9], solar cells [5,11,12], and laser optical media [2,6,13,14,15,16,17]. Conjugated materials have appealing photophysical properties for the implementation of optical devices, namely, large stoke shift emission, excellent gain, strong absorption, high-quality factor, and the ability to produce tunable lasers [18,19,20].

In practice, the extrapolation of the electrical and optical properties of conjugated polymers throughout the length of the chain is very difficult. The properties of conjugated materials do not change by increasing the conjugation length beyond the effective conjugation length (ECL). Therefore, conjugated oligomers (COs) can have similar or better properties by controlling the conjugation length and limiting the number of separations [21,22].

COs are a special class of materials that have the advantage of both a small dye laser and conjugate polymer laser materials and can have excellent chemical stability. COs have a low molar mass, a uniform molecular structure, and consist of more than one repetition unit but less than 20 units. These materials have the advantage of easy processing with the ability to be deposited as a thin film using inexpensive techniques such as spin coating. Intensive research efforts have focused on laser action from COs in a liquid state and a thin film, especially through optical pumping. Therefore, the stimulated emission and gain can be studied without the difficulties related to current injection, electrode incorporation, and charge transport. COs dissolve in a wide range of solvents and form very high-quality films [23]. These [24] materials have compatibility with a wide range of materials, which render them suitable for blended, composite, and hybrid materials. M. S Park et al. studied the wet-etching of polymer/fullerene blend films. The incorporation of oligoelectrolytes into membranes changed the optical and electrical properties of the resulting membranes [25].

Many research groups have studied mirrorless lasers and lasers with cavity resonators from CO thin films and solutions. Dario Pisignano et al. studied amplified spontaneous emission in spin-coated films of a substituted thiophene-based oligomer (T5oCx) on a glass substrate. A very low threshold and large gain cross section for line narrowing due to ASE were achieved. The laser action of a T5oCx film using a plane-concave resonator was observed. The optical excitation source was the second harmonic (390 nm) of a Kerr lens mode-locked Ti: sapphire laser (390 nm, 150 fs, and a repetition rate of 1 kHz pulses at 780 nm). The results confirmed that the T5oCx material is an excellent candidate for organic laser materials [26].

M. Anni et al. demonstrated a random laser with an 8° full width half maximum (FWHM) divergence of the unsubstituted quinquethyenil S,S-dioxide(T5oCx) film, which has a thickness of 450 nm. The film was cast on a glass substrate in a chloroform solution using the spin coating method. The pump source was of the 3rd harmonic (355 nm) from a Nd:YAG laser system [27]. In 2010, research group explored the photophysical properties and organic thin-film lasers of the truxene oligofluorene (T3) oligomer. ASE was achieved and peaked at 439 nm with a low threshold of 2.1 kW/cm^2^. The truxene oligofluorene T3 material exhibits a net gain of up 38 cm^−1^ and has solid-state PLQY of 86 ± 10%. Moreover, these authors reported a broad tunability of 51 nm of T3 in the deep blue/blue region of the spectrum using a DFB laser design [28]. Low ASE thresholds of 30 nJ pulse^−1^ and high maximum net gain coefficient of 55 cm^−1^ were achieved in oligofluorene-pyrene starburst thin films. The film thickness varied from 150 to 200 nm, and the optical pump wavelength 375 nm was provided by a Q-switched Nd^3+^: YAG laser [29].

In 2014, the morphology, absorption, photoluminescence, and amplified spontaneous emission (ASE) properties of oligofluorene thin films were studied. The results indicate that the oligofluorene derivatives are a promising material for optically pumped solid-state lasers [30]. Our group produced a mirrorless laser from a conjugated oligomer (CO) 9,10-Bis[(9-ethyl-3-carbazoyl)-vinylenyl]-anthracene (BECVA), which peaked at 500 nm in the green region via the transfer energy process [31]. In 2015, a tunable, solution-processable distributed feedback laser was designed using a carbon-bridged oligo(p-phenylenevinylene) oligomer by Marta Morales-Vidal et al. [32]. A thin-film organic laser (TFOLs) was accomplished using perylene dyes and p-phenylenevinylene (PV) oligomers [33].

In this study, we demonstrate the photoluminescence, absorption and mirrorless laser properties of the CO BECV-DHF thin film under transverse excitation (3rd harmonic Nd: YAG pulsed laser). The results show that CO BECV-DHF in thin films has a low energy threshold of 0.65 mJ and produces an ASE at 482.5 nm with a full width at half maximum (FWHM) of 6.8 nm. Time-resolved spectroscopy of the oligomer thin film provides information with respect to the wavelength (nm), intensity (a.u.) and time (ns) on the *X*, *Y*, and *Z* axes, respectively. The laser-induced fluorescence (LIF) and ASE of the CO BECV-DHF showed evidence for damping of the first band due to H-type aggregation. The waveguiding nature of the film produces very efficient ASE in a pure oligomer film. However, this could be the first report of time-resolved spectroscopy (TRS) of ASE oligomers in thin films.

## 2. Materials and Methods

**Figure 1:**
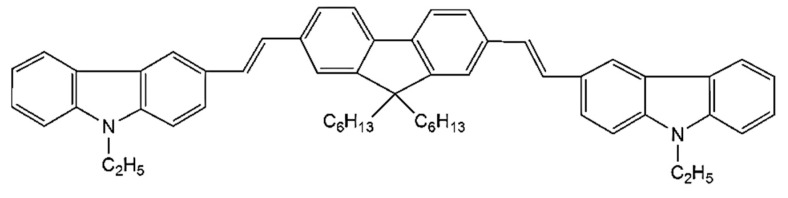
The molecular structure of 1,4-bis(9-ethyl-3-carbazo-vinylene)-9,9-dihexyl-fluorene (BECV-DHF).

**Figure 2:**
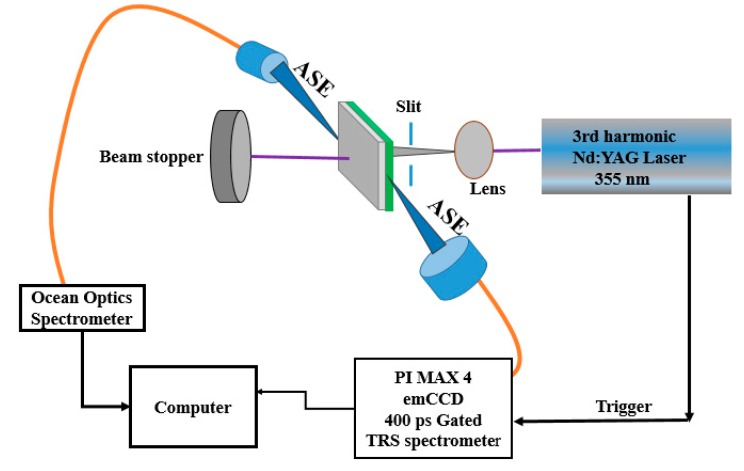
Laser experimental setup for the CO films.

The CO BECV-DHF was purchased from American Dye Source, Inc. (Quebec, QC, Canada) and had a molecular weight of 773.12 g mol^−1^ with a molecular structure, as shown in Figure 1. Laboratory-grade solvents such as chloroform were purchased from Sigma Aldrich, St. Louis, MO, United States. A Perkin Elmer Lambda 950 spectrophotometer (Llantrisant, United Kingdom) was used to record the absorption spectra over a wide range (200–1000 nm). The fluorescence spectra were measured using a spectrofluorometer (LS 55, from the same company) over a scan range of 200 to 1000 mm [34]. The laser pulse (a 5 ns laser of 3rd harmonic (355 nm) from Nd:YAG) (Brilliant B, Quantel, Les Ulis, France) was focused by a cylindrical lens with a 5 cm focal length and fixed horizontally; therefore, the laser pulse was concentrated to a strip of 1 mm × 1 cm (length × width), as shown in Figure 2. The thin film samples were placed away from the focal point to avoid laser ablation of the active media. The samples produced LIF or ASE based on the pump energy, which was collected using fiber optic cables at both sides. One fiber was connected to the Ocean optics spectrograph with a wide range of 200 to 1100 nm (Maybachstrasse, Ostfildern, Germany), and another was connected to the PI. MAX 4 ultrafast-gated emCCD ultra cooled camera, with an Acton spectrograph (Princeton Instruments, Trenton, NJ, USA), which had a gate of less than 400 ps.

**Figure 3:**
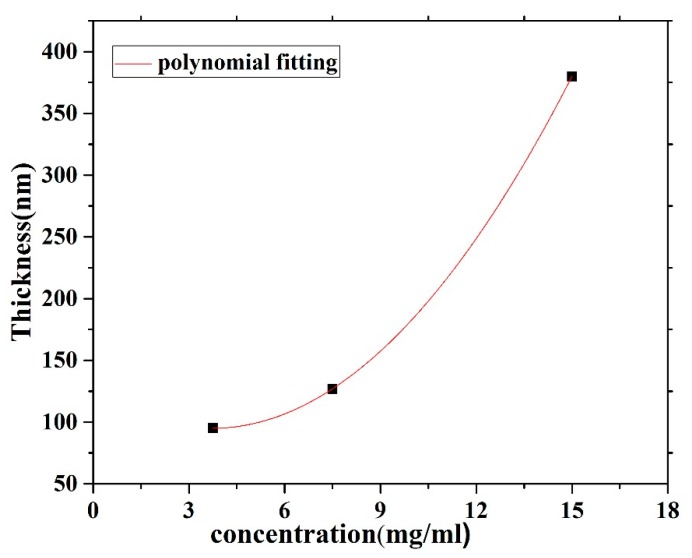
Film thickness versus the solution concentration for CO BEVC-DHF deposited by spin coating at 2000 rpm.

The CO BECV-DHF films were cast by spin coating onto quartz and silicon substrates from chloroform solutions at different concentrations (15, 7.5 and 3.75 mg/1 mL). Spin coating speeds of 2000 and 3000 rpm for 20 s were employed to acquire neat films with different thicknesses. A surface profiler Bruker DekTakXT was used to determine the thickness of the films. The thickness of the CO film was varied by varying the concentration of the film, whereas the spin speed was maintained at 2000 rpm as well as 3000 rpm. The result shows that the thickness increased with respect to the concentration, as presented in Figure 3. A Veeco Multimode Quadrax MMAFM (Bruker; Billerica, MA, USA) was used to record the Atomic Force Microscopes (AFM) image measurements at room temperature under an ambient atmosphere. The morphology and structure of the film’s surfaces were revealed using a JSM-7600F scanning electron microscope (JEOL, Peabody, MA, USA).

## 3. Results and Discussion

### 3.1. Atomic Force Microscopy (AFM)

**Figure 4:**
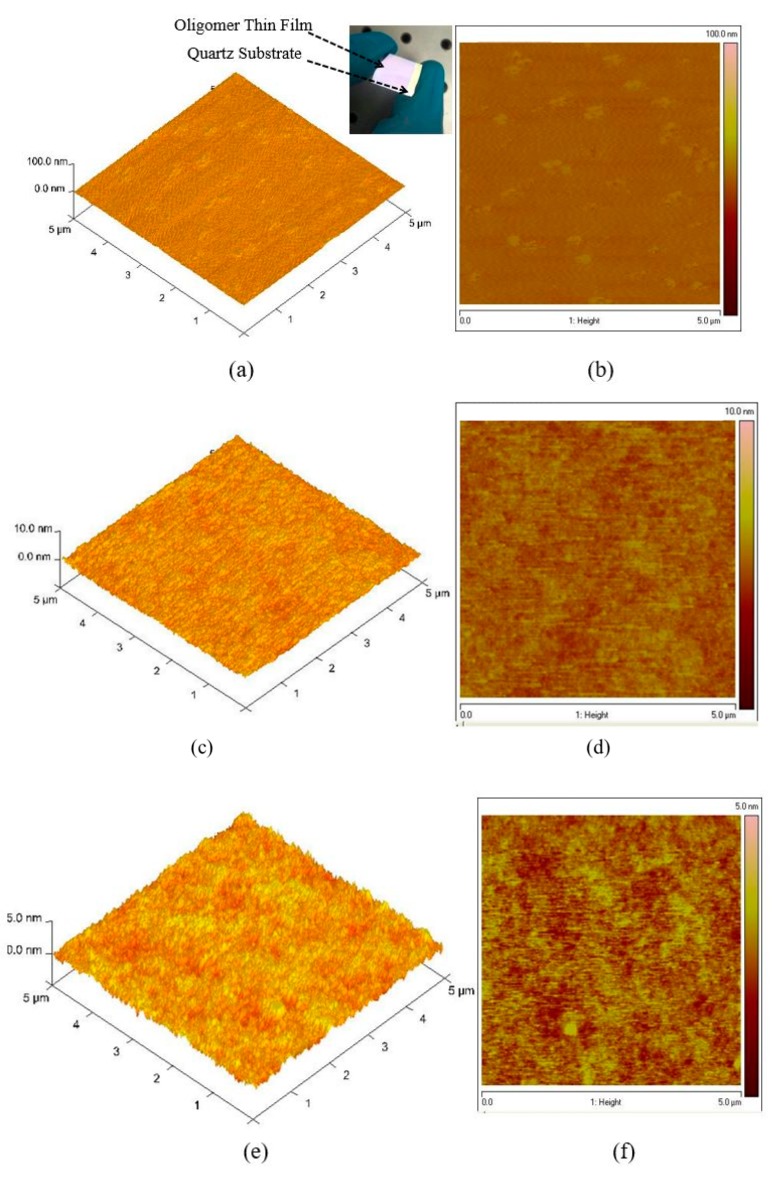
Atomic force microscopy (AFM) images of the oligomer at thicknesses of 270, 88, and 70 nm. (**a**,**c**,**e**) are 3D surface of film with thickness 270, 88 and 70 nm, similarly (**b**,**d**,**f**) are 2D morphology of film with thickness 270, 88 and 70 nm respectively.

The topological image of the oligomer thin films was recorded using AFM, as presented in Figure 4a–f. Figure 4a,b shows the 3D morphological structure of the film coated at 3000 rpm at 15 mg/mL. The thickness of the sample was measured using a profilometer, which showed uniform film formation, and the thickness was 270 nm. The surface was extremely smooth. The AFM images show that the root-mean-square (RMS) roughness of the samples increases with increasing surface roughness maximum (Rmax) = 8.3, root mean square average (Rq) = 1.23 and arithmetic average (Ra) = 0.989 nm). Figure 4c,d shows a high surface profile in which the lowest pit was 4 nm deep and the node was 2 nm. The solution concentration was 7.5 mg/mL, and the thickness was 88 nm. The measured surface roughness was Rmax = 2.07, Rq = 0.392 and Ra = 0.312 nm. The morphology of the thin-film samples made from a concentration of 3.75 mg/mL and a thickness of 70 nm is presented in Figure 4e,f. The measured surface roughness was Rmax = 1.47, Rq = 0.283, and Ra = 0.224 nm. The AFM results reveal that the samples have a very smooth surface and that the roughness was very low, which indicates that the oligomer films can be self-assembled and it suitable for various devices such as solar cells [35,36], Organic Light Emitting Diodes (OLEDs) [37] and lasers [38]. The low amount of surface defects is ideal for a laser material because they facilitate low speckle formation and very low scattering loss and could become a good planar waveguide that allows surface laser emission. Therefore, thin films of CO BECV-DHF could become a vertical-cavity surface-emitting laser.

### 3.2. Scanning Electron Microscope Analysis

**Figure 5:**
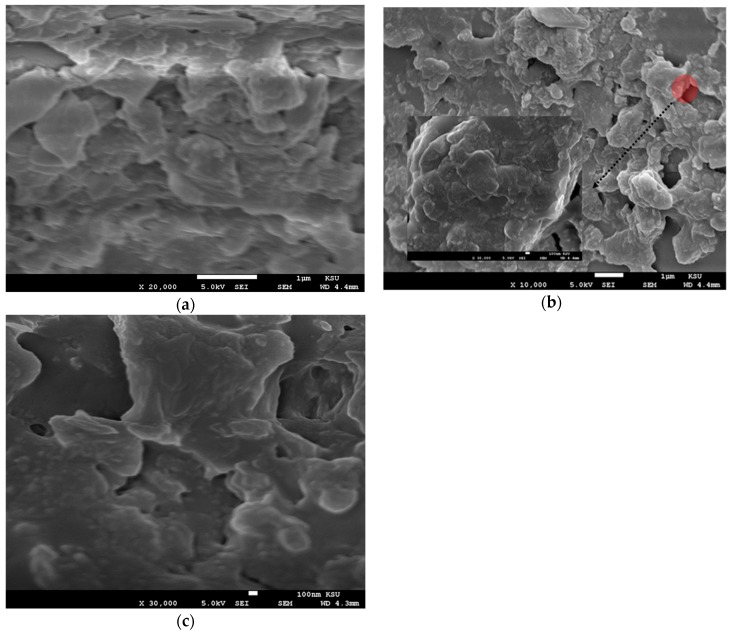

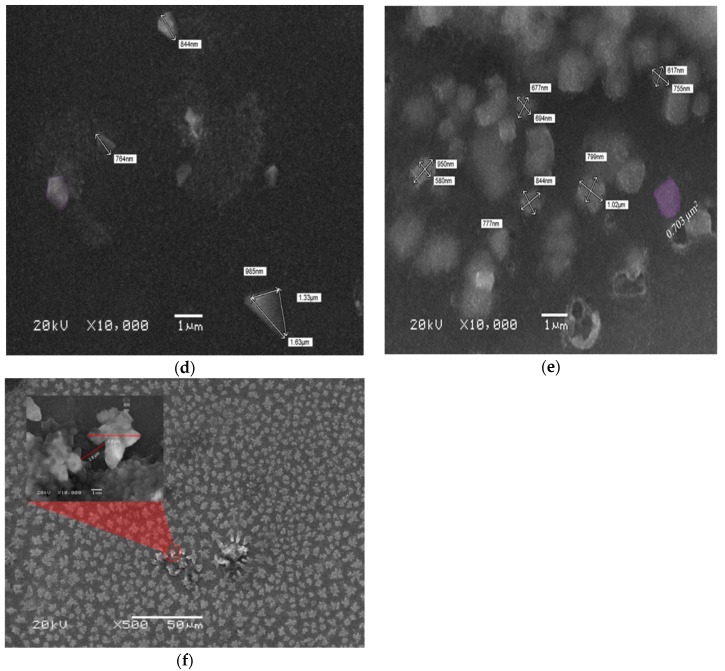
(**a**–**c**) SEM images of the oligomer at thicknesses of 70, 88, and 270 nm and speed 3000 rpm. (**d**–**f**) SEM images of the oligomer at thicknesses of 95,127, and 380 nm and speed 2000 rpm.

SEM analysis was performed on the CO thin films of different thicknesses. The films were coated using solutions at different concentrations of 15, 7.5, and 3.75 mg/mL via different spin speeds of 2000 and 3000 rpm. The samples were found to have a very low conductivity; therefore, all samples were coated with platinum for 40 s. This form of a small top layer does not affect the morphology of the films. All the films were studied using SEM at the different concentration (3.75, 7.5, and 15 mg/mL) and spin speed of 2000 and 3000 rpm, as presented in Figure 5. The images showed the formation of flakes with amorphous and random shapes as in Figure 5a–c. This finding indicates that at a high spin speed, the solution droplet expanded and dried faster, so the oligomer does not form a crystal structure.

In Figure 5d, the sample with a concentration of 3.75 mg/mL and thickness of 95 nm showed triangular and pentagonal crystal formations. The crystals were separated by a large distance, which could be due to the low concentration. The sample made with 7.5 mg/mL and thickness 127 nm at the same spin speed showed mostly squared crystals, and the crystals were close to each other, as presented in Figure 5e, in addition Figure 5f shows the SEM images of films at a concentration of 15 mg/mL and thickness 127 nm. The images revealed the formation of flower-like crystals, the separation distance was very small, and the distribution of crystals was homogenous. Self-assembly of a CO is largely depend on alkyl chain, like in BECV-DHF the contribution towards the morphology of film is mainly due to diphenyl group in 9,9-dihexyl-9H-fluorene and also BECV-DHF contains one imide position at each BECV-DHF which plays a key role in electron mobility could improve the performance of devices [39].

### 3.3. Absorption and Photoluminescence Properties of the Oligomer Film

**Figure 6:**
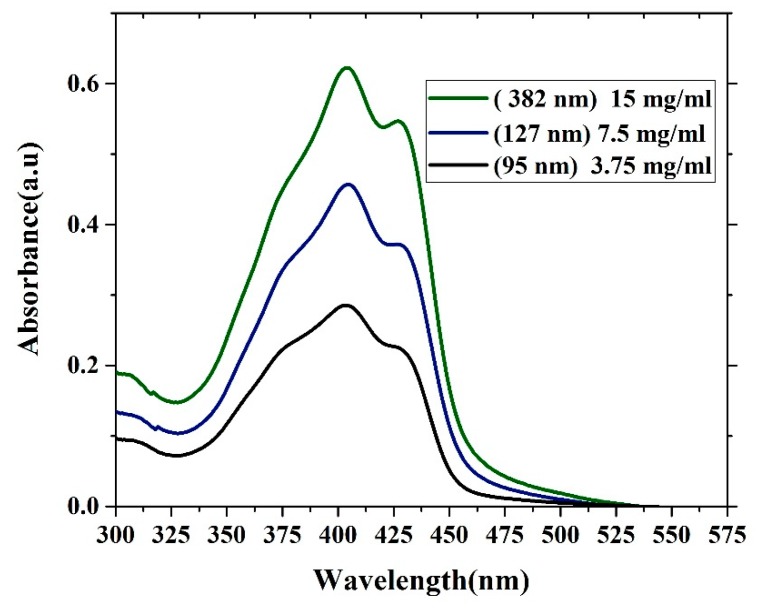
Absorption spectra of the CO BEVC-DHF samples with different thicknesses (380 nm, 127 nm, and 95 nm).

The absorption properties of oligomer thin films with different thicknesses are presented in Figure 6. The absorption spectra contain three features. One shoulder located at approximately 375 nm is due to the vibronic transition S0-S2 (V1 monomer) transition, and the main peak located at 403 nm is due to the S0-S1 (V2 monomer) transition. Additionally, the peak at approximately 450 nm is due to aggregation mostly referred to as dimerization (D-peak). The ratio of (V1/V2) decreases as the thickness increases because of the damping of (V1) at the low-thickness film due to a reduction in the reabsorption and a decrease in the degree of freedom. In contrast, the ratio of (D/V2) increases with increasing thickness, which could be due to the formation of more dimers. The optical density of the samples increases as the thickness increases. This result could be due to an increase in the surface reflection, and the attenuation of the wavelength could be due to increased scattering. The FWHM of all the samples was approximately 88.5 nm. The ratios of (V1/V2) and (D/V2) are given in Table 1.

### 3.4. Photoluminescence (PL) Spectra of the Oligomer Thin Films

**Figure 7:**
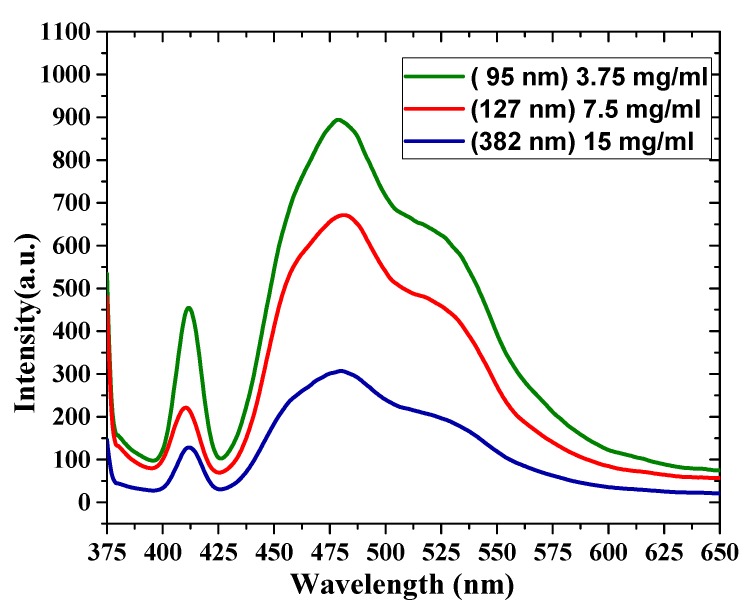
Photoluminescence (PL) spectra for different film thicknesses (380, 127 and 90 nm).

The PL spectral properties of oligomer thin films are shown in Figure 7. The PL was recorded at room temperature at an excitation wavelength of 355 nm. This wavelength was chosen to coincide with the 3rd harmonic of the Nd:YAG laser, which was used as a pump source. The PL of the thin-film samples has broad emission between 400 and 550 nm, and there are four features in the PL spectra. The first peak was located at 411 nm (V0), the second peak was located at 455 nm, and the third peak was located at 480 nm. Additionally, there was a shoulder at 523 nm. The FWHM of the samples decreased as the thickness increased, with values of 93, 96, and 99 nm. The ratios between these different peaks of all the samples are presented in Table 2. The PL emission depends on and is inversely proportional to the film thickness, due to reabsorption.

### 3.5. Band Gap Analysis

**Figure 8:**
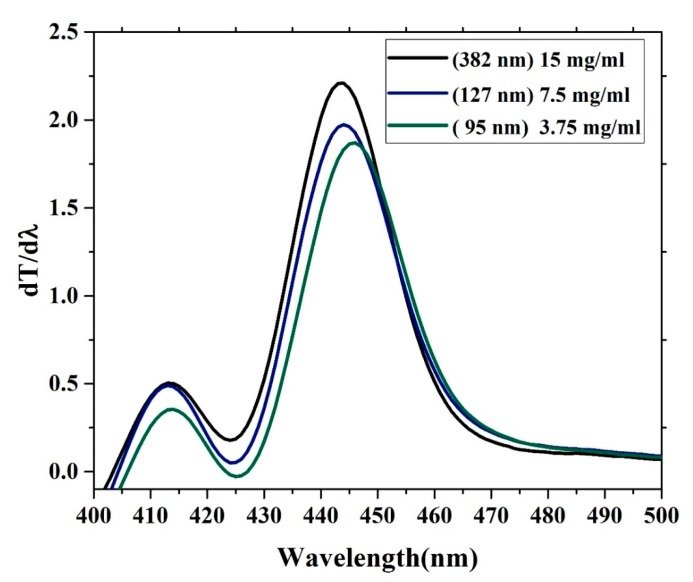
Change in transmission versus wavelength first-order derivatives of the transmission concerning the wavelength.

**Figure 9:**
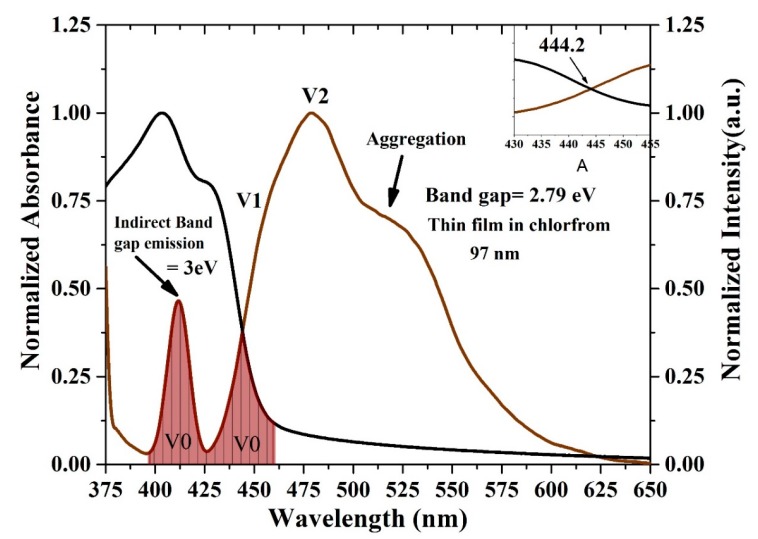
Optical energy gap by crossing the absorption and fluorescence spectra of the CO thin film. The black and brown curves are normalized absorption and fluorescence spectra, the area fill with brown color indicates the spectral overlap between the absorption and fluorescence spectra, attributed to V0 (0-0) band.

The oligomer showed that an increase in the thickness reduces the transmission. The energy gaps (*E*g) of the CO were obtained according to the following relation (*E*g = *hc*/*λ*max), where *h* is Planck’s constant, *c* is the light speed, and *λ*max is the wavelength in the maxima of the derivative curve. The sharp peak at *λ* = 445 nm indicates that *E*g = 2.78 eV, which is directly attributed to the vibration transition S0-S1 (V1) of the monomer as shown Figure 8. The band gap shifts a small amount toward red when the thickness is reduced. Another minor peak at approximately *λ* = 413 nm was due to the transmission between S0-S0 (indirect band gap), which is the overlapped vibration band (V0, electronic state 0-0) of the monomer with approximately *E*g = 3 eV. Furthermore, no band is shown in the longer wavelength region; however, there is a long tail, which indicates aggregation in the ground state. Although new peaks appeared in the first-order derivatives of the transmission concerning the wavelength (d*T*/d*λ*) spectra at 413 nm, the band gap is considered to be 445 nm because it is in the longer wavelength region beyond which there is no absorption or transmission peak due to electronic transitions. Therefore, the peak at approximately 445 nm is not chosen because it has a higher amplitude. It is also possible to find the *E*g of the CO films by crossing the absorption and fluorescence spectra as shown in Figure 9. The peak at 413 nm in the shorter wavelength region was also compared to the intersection of the fluorescence and absorption spectra, which is the indirect band gap due to the overlap as presented in Figure 8. The results revels that the band gaps are almost the same.

### 3.6. ASE (Mirrorless Laser) from CO BECV-DHF Films

ASE and lasing action have been achieved from conjugated polymers and oligomers in much research work [13,40,41]. A thin film of thickness 380 nm was pumped with different energies, and the film was kept beyond the focal point of a cylindrical lens to avoid the photo-ablation process destroying the film. The ASE emerged on both sides of the excitation strip. The FWHM of the ASE was only 6.8 nm. The oligomer film can be compared to the extremely high concentration solution, where the oligomer has three absorption peaks. Furthermore, suitable concentrations produce ASE at approximately 466.5 nm, whereas in the thin film, the ASE shifts to a longer wavelength of 482.5 nm.

**Figure 10:**
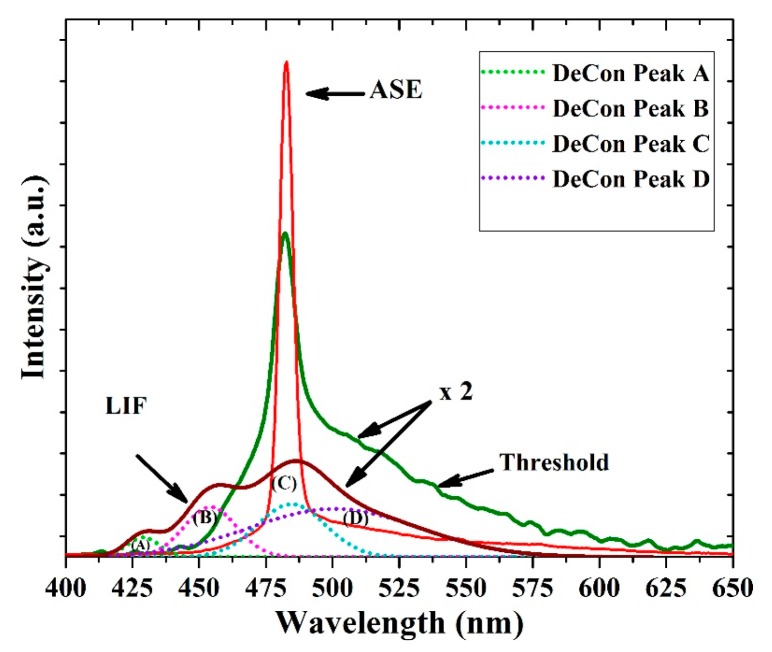
Amplified spontaneous emission (ASE), threshold, and laser-induced fluorescence (LIF) spectra of the CO film with a thickness of 380 nm and a concentration of 15 mg/mL. The Figure 10 also shows deconvolution (DeCon) of LIF spectra, where the light green (A), Pink (B), sky blue (C) and violet (D) bands are ascribed to V0, V1, V2 and dimer bands.

Figure 10 shows laser-induced fluorescence spectra with three distinct peaks at 425, 455 and 485 nm along with one shoulder at approximately 525 nm. The FWHM of the LIF was at 67.3 nm. The deconvolution of the LIF spectra exhibits four peaks, as shown in Figure 10. The first peak (A) at 425 nm is due V0 shifted, the second peak at 455 nm corresponds to V1 and the 485 nm is V2 peak, at approximately 496 nm. The aggregation peak which due to the dimer and the tail peak is located at approximately 520 nm, which is due to the reabsorption of all bands such as (A), (B) and (C) in Figure 10; therefore, it has a large overlap from all deconvoluted bands.

When the pump energy was increased to 0.65 mJ, the spontaneously emitted photons were guided by the oligomer thin film and amplified by stimulated photons into a spectrally narrow, distinctively shaped band appearing at 482 nm with an FWHM of 15 nm. As the pump energy further increased to more than 1.1 mJ, the ASE appeared and peaked at 482.5 with an FWHM of 6.8 nm. The ASE peak likely corresponds to the V2 transition. However, the aggregation process produces a broad LIF band along with a narrow ASE peak, and the LIF broadband is present in the spectra; this is typical for a thin film due to high reabsorption and emission from the dimer. The spontaneously emitted photo from the V2 band is waveguided and induced more stimulated emission inside and on the surface of the film, due to very smooth surface evident from AFM image gives rise to the low threshold mirror less light amplification [38].

**Figure 11:**
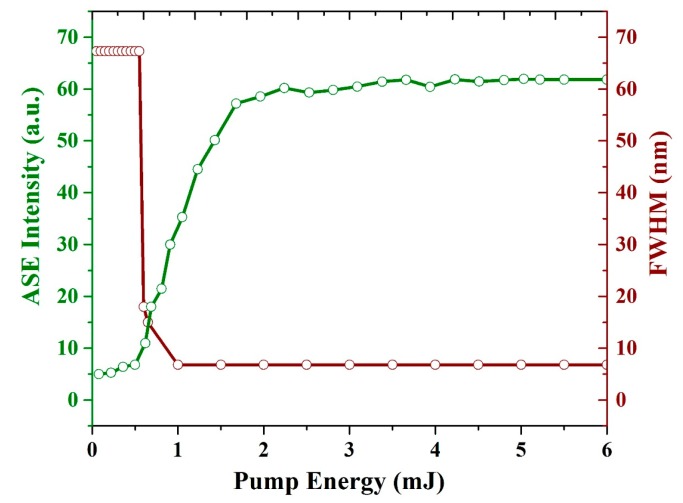
Relationship between the pump energy, intensity, and FWHM of the ASE of the CO film with a thickness of 380 nm and a concentration of 15 mg/mL. The green line indicates the ASE intensity (a.u.) and brown line indicates FWHM (nm).

Figure 11 illustrates the spectral narrowing (FWHM) and ASE intensity as a function of the pump energy. The threshold of the CO film is 0.65 mJ. The intensity starts increasing, and more importantly, the FWHM of the spectrum is reduced from 67 to 15 nm. As the pump energy increased further to 1.1 mJ, the FWHM reached 6.8 nm, and the ASE intensity increased up to the saturation level of the detector as the pump energy increased beyond 5 mJ.

### 3.7. TRS of the CO

**Figure 12:**
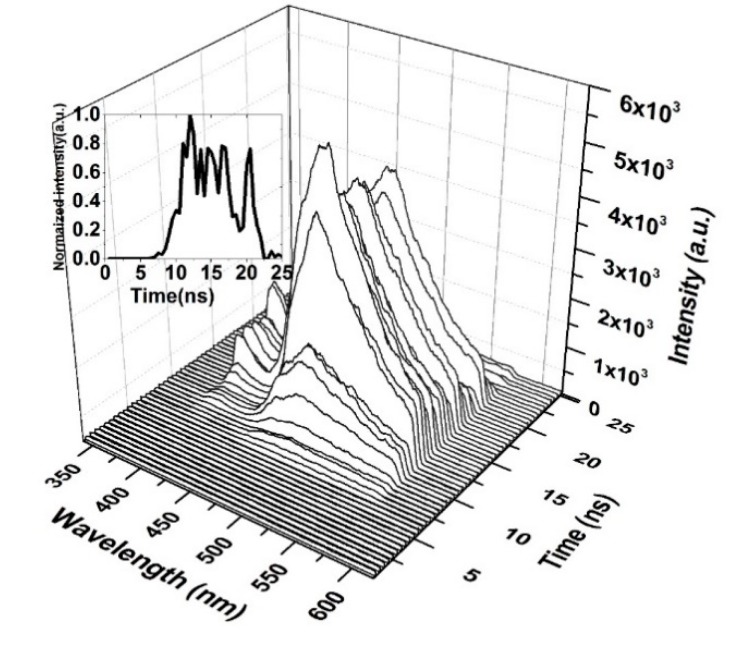
Time dynamics of CO thin-film LIF in chloroform at a pump energy of 0.55 mJ and a concentration of 15 mg/mL. The subfigure is the Z slice at 483 nm, which gives the intensity (a.u) vs time (ns) profile of the LIF peak at approximately 485 nm.

Figure 12 shows the time-resolved spectroscopy of the oligomer thin film when the pump energy was 0.55 mJ. Thin films are unable to produce ASE and produce only LIF, which is broad. The fluorescence of the V2 band starts first, as it dominates the V.B in the thin film. The fluorescence tail continues up to 600 nm (cut in the shown figure due to gratings selection, and the range of study was 200 nm). The first vibration band V1 appears only after 5 ns, which could be due to the damping effect that occurred because of H-dimer aggregation. The intensity of LIF rapidly is increased and is maintained for 4 ns, and a blink occurred due to the saturation of the thin film at approximately 18 ns, as shown in the inset (time evolution of the LIF process).

**Figure 13:**
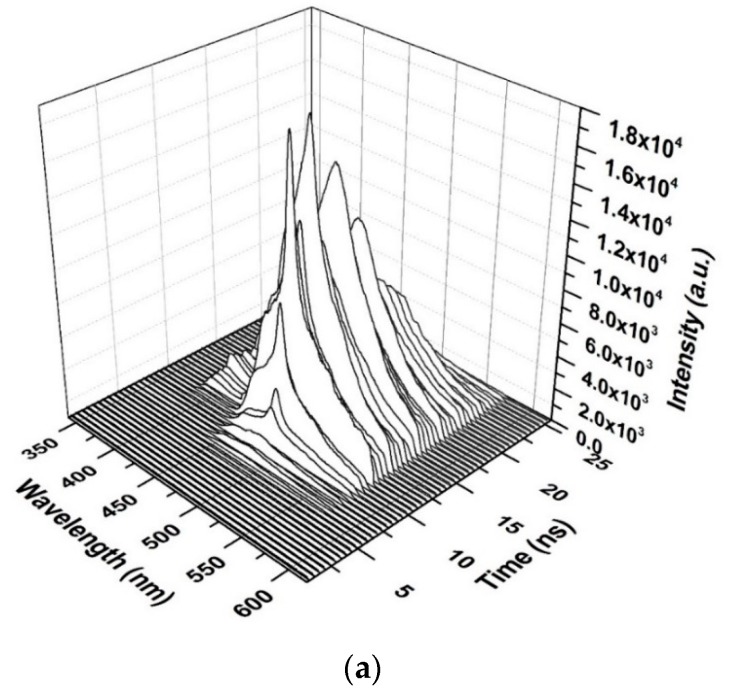

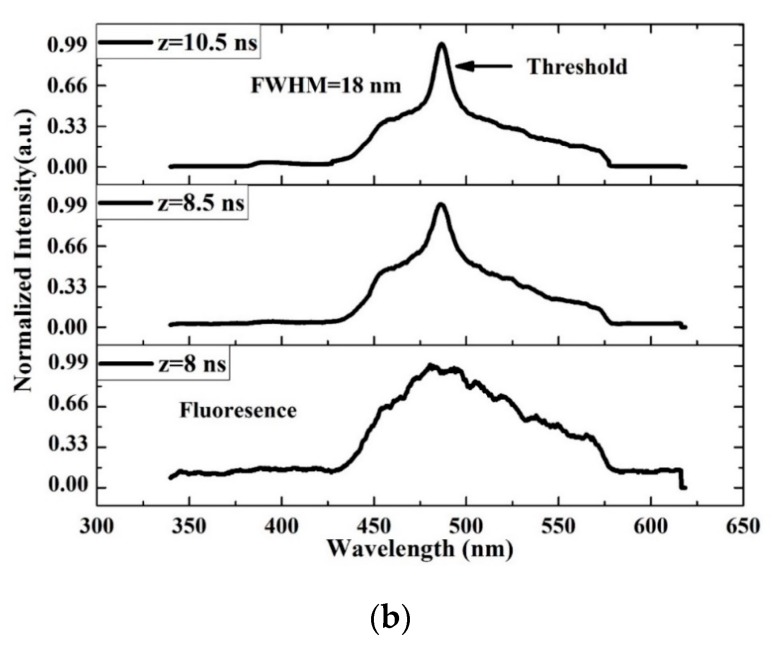
Time dynamics of the CO thin film (**a**) threshold (**b**) time profile of threshold in chloroform at pump energy of 0.65 mJ and a concentration of 15 mg/mL.

The TRS of the threshold pumping is shown in Figure 13. The pump energy is 0.65 mJ, and the threshold ASE occurred at 10.5 ns. The LIF is a broad band with ASE emerging on its top, where the LIF peak is approximately 482 nm. The important events are given in Figure 13b. The results show that at 8 ns, the LIF appears and starts narrowing at 8.5 ns, and at 10 ns, the threshold of ASE takes place with an FWHM of 18 nm and more intensity.

**Figure 14:**
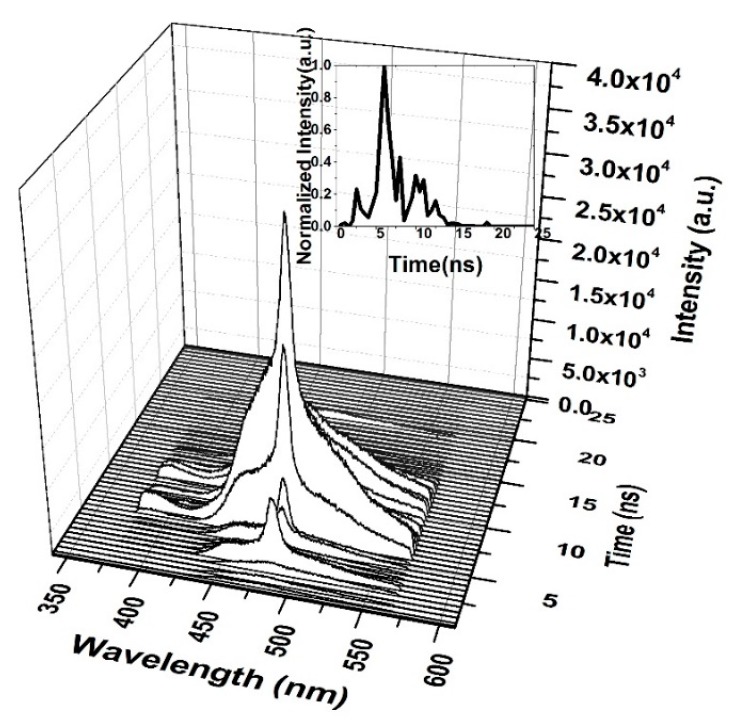
Time dynamics of CO in chloroform at pump energy of 3 mJ and a concentration of 15 mg/mL. The subfigure is the Z slice at 482 nm, which gives the intensity (a.u) vs time (ns) profile of the ASE peak at 482.5 nm.

When the CO thin film was pumped at a 3 mJ pulse energy, the TRS spectra of the ASE were recorded, as displayed in Figure 14. Therefore, the ASE starts after 1 ns of the LIF, and the pulse of the ASE is very short. The saturation of absorption occurs at 5 ns, and therefore the ASE pulse is produced after 5 ns, at a very low intensity. This finding is compared to the peak intensity shown in the inset of Figure 14. The ASE is at the top of the LIF because of spectral broadening due to the reabsorption of the emitted ASE.

A high pump energy (5 mJ) was applied to the film; the ASE occurred in 1.5 ns from fluorescence. The ASE appears to rise and fall faster when the trailing side of the ASE is absent. The other band also appears due to the high pump energy.

**Figure 15:**
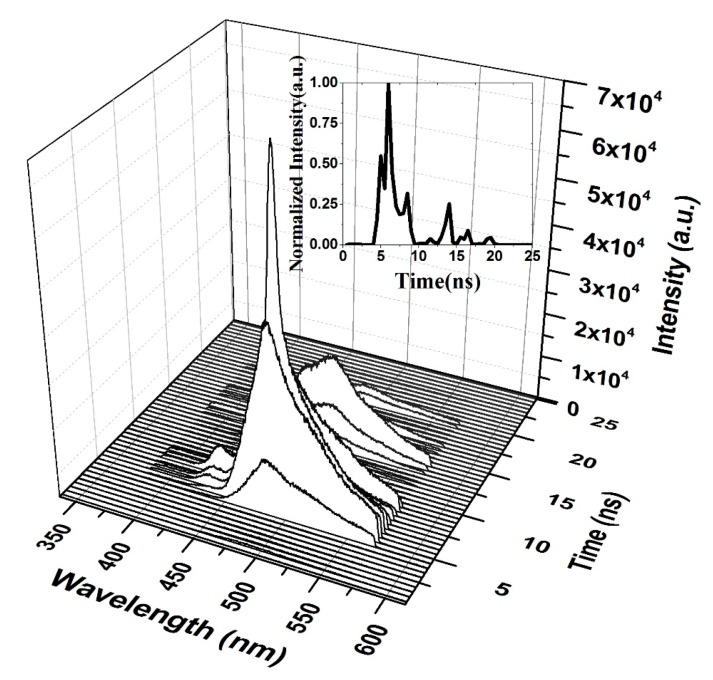
Time dynamics of the CO thin film at 15 mg/mL and at a high pump energy (5 mJ). The subfigure is the Z slice at 487 nm, which gives the intensity (a.u) vs time (ns) profile of the ASE peak at approximately 483 nm.

The oligomer films have a very large number of self-assembled molecules that are close to each other. This effect reduces their degree of freedom (vibration). In addition, the excited oligomer molecules have many trapping states and more decay paths when compared to the oligomer solutions. This behavior leads to saturable absorption. That is, when all molecules are in the excited state and more photons are still available, the molecules in the excited states absorb and transport to higher excited states. Therefore, the oligomer films produce a pulse width with a shorter lifetime than the pump laser’s lifetime. The ASE of the oligomer exhibited a longer lifetime for a low pumped energy of 1.25 mJ, and when the pumped energy was increased, the total photon flux available for molecules is high; therefore, saturation takes place. This process induces a blink (absorb stopping ASE action). These spontaneously emitted photons are waveguided, produce stimulated photons, and therefore, a short pulse is produced, as shown in Figure 14 and Figure 15.

### 3.8. Stability of ASE from BECV-DHF films

**Figure 16:**
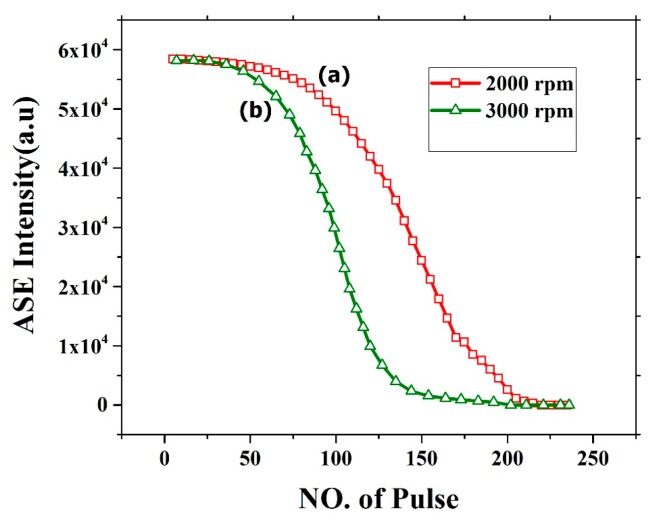
ASE stability of CO films with different thickness at different speed (**a**) 2000rpm and (**b**) 3000 rpm with 250 pulse and energy 2.5 mJ.

The oligomer film process demonstrated very good ASE but suffers from photochemical and photomechanical instability. Figure 16 shows the photochemical stability of the CO thin film thickness of 382 nm (2000 rpm) and 270 nm (3000 rpm). The stability of CO for 2000 rpm film was relatively stable for 100 pulses at pump energy of 2.5 mJ. After approximately 120 pulses, the ASE intensity deteriorates and falls completely to zero at approximately 200 pulses. The film was completely etched, and the ASE was absent as shown in Figure 16a. This effect is mainly due to the laser ablation of the film. similarly, for 3000 rpm the stability was less due to low thickness as shown in Figure 16b. The stability of the film can be improved by introducing a protective epoxy layer or transparent metal coating on top of the CO.

**Figure 17:**
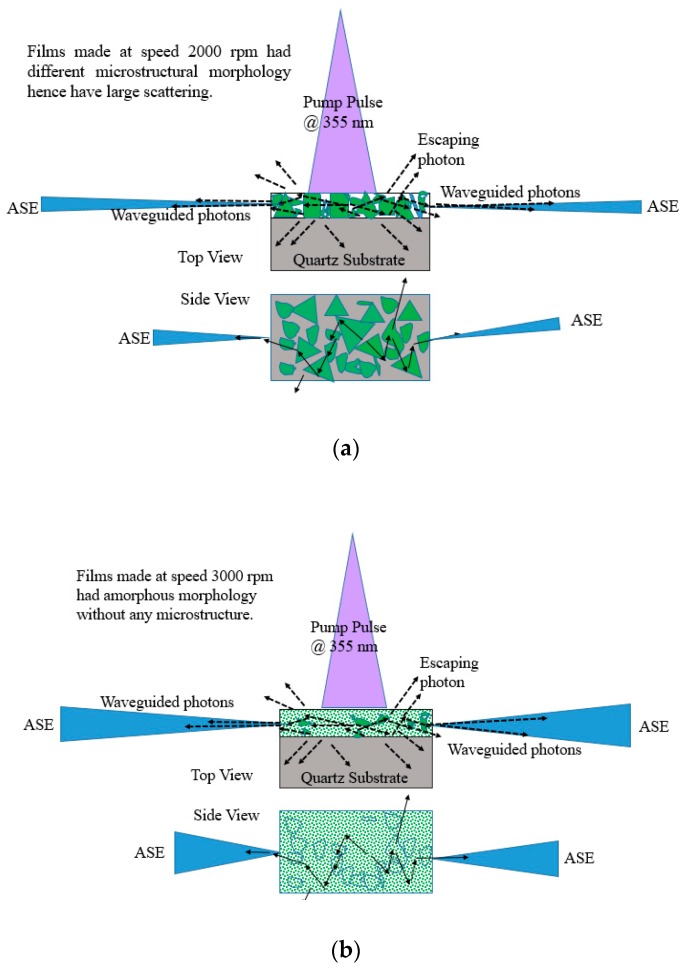
Waveguiding nature of the CO thin films for (**a**) 2000 rpm and (**b**) 3000 rpm. The green shapes in Figure 17a represent different oligomer crystal structures (for 2000 rpm) and green wave in Figure 17b represents amorphous structure. The blue shapes with in the film represent the grain boundaries. The blue cone or triangle in Figure 17a,b represents the ASE beam.

The ASE produced from these films depend on the morphology, the film with thickness 380 nm made from 2000 rpm spinning had microstructures and defects as shown in SEM images Figure 5 due to which they have good stability and produce ASE due to waveguiding in the microstructures as shown in Figure 17a. However, for 3000 rpm, the films become amorphous; they produce ASE, but have low photochemical stability when compared to 2000 rpm made films as presented in Figure 17b.

## 4. Conclusions

Oligomer thin films on top of quartz were deposited using a spin coating technique. These films have different thicknesses of 380, 127, and 95 nm. Under a suitable pump energy excitation, the film with a thickness of 380 nm produces ASE at 482.5 nm, which is a completely different wavelength than the oligomer in liquid (466.5 nm). The new ASE peak at 482.5 nm is ascribed to the V2 band of the oligomer; therefore, ASE from the oligomer V2 vibrational band is reported. The TRS showed that the primary vibration band (V1) appears much later due to the vibration damping effect, and ASE occurs on top of the broadband LIF, due to spectral broadening caused by the high concentration of the oligomer in the thin film, which induces reabsorption and scattering. Nevertheless, this could be the first report of the TRS of ASE oligomers in thin films.

## Figures and Tables

**Table 1:** The absorbance ratio of the different peaks of the CO films.

	Absorbance (Units)			Ratio (%)	
Samples (Concentration/Thickness)	V1 375 nm	V2 403 nm	D 428 nm	R (V1/V2) %	R (D/V2)%
3.75 mg/mL 95 nm	0.227	0.285	0.244	78.59	79.64
7.5 mg/mL 127 nm	0.335	0.455	0.370	73.62	81.31
15 mg/mL 380 nm	0.449	0.622	0.546	72.18	87.8

**Table 2:** The photoluminescence (PL) ratio of the different peaks of the CO films.

Samples (Concentration/Thickness)	PL intensity (a.u)				Ratio (%)		
	V0 at 411 nm	V1 at 455 nm	V2 at 480 nm	Shoulder at 523 nm	(V0/V2)	(V1/V2)	(Sh/V2)
3.75 mg/mL 95 nm	452	653	892	629	50.07	73.2	70.5
7.5 mg/mL 127 nm	220	506	671	465	32.8	75.4	69.3
15 mg/mL 380 nm	127	234	307	200	41.4	76.2	65.1
